# Invasive fungal infection by *Saprochaete capitata* in a child with bone marrow aplasia

**DOI:** 10.17843/rpmesp.2022.393.10955

**Published:** 2022-09-30

**Authors:** Julio Maquera-Afaray, Camila Escajadillo-Vergara, Jeanpiero Chire-Mercado, María Paula Durand

**Affiliations:** 1 Instituto Nacional de Salud del Niño San Borja, Lima, Peru. Instituto Nacional de Salud del Niño San Borja Lima Peru; 2 Universidad Privada de Tacna, Tacna, Peru. Universidad Privada de Tacna Universidad Privada de Tacna Tacna Peru

**Keywords:** Invasive Fungal Infections, Mycoses, Fungi, Antifungal Agents, Aplastic Anemia

## Abstract

*Saprochaete capitata* is a rare cause of invasive fungal infection in immunocompromised patients with high mortality and antifungal resistance. We present the case of a 5-year-old boy with bone marrow aplasia, who underwent hematopoietic stem cell transplantation (HSCT) and presented persistent febrile neutropenia, abdominal pain, appearance of maculopapular lesions on the skin, and impaired renal function. The presence of *S. capitata* was identified by blood culture from a central venous catheter. This invasive fungal infection is rare but emergent and life-threatening, especially in immunocompromised patients with persistent febrile neutropenia and prolonged use of invasive devices such as central venous catheters.

## INTRODUCTION

*Saprochaete capitata* (anamorph)/*Magnusiomyces capitatus* (telemorph), previously known as *Geotrichum capitatum* or *Blastoschizomyces capitatus*, is a yeast-like fungus that belongs to the Dipodascaceae family, Saccharomycetales order ^1^. This microorganism is found in the environment, especially in soil, and can colonize the skin, respiratory and gastrointestinal tract in humans ^2^. Likewise, *S. capitata* is an opportunistic fungus that mainly affects immunocompromised patients with oncohematological disease and prolonged neutropenia ^3^.

The incidence rate of fungemia by *S. capitata* is low, being considered a rare cause of invasive fungal infection, with reports of sporadic cases and some nosocomial outbreaks described in different countries ^1^ ^,^ ^4^; nevertheless, in the last decades the number of cases of *S. capitata* has increased, due to the greater use of immunosuppressive therapies and identification of the causal agent using better microbiological diagnostic methods ^2^ ^,^ ^5^. However, the mortality described for *S. capitata* is still high, fluctuating between 40 to 75% of the cases ^1^ ^,^ ^6^.

In this article we report the first case of invasive fungal infection by *S. capitata* in Peru, which occurred in an immunocompromised child with bone marrow aplasia who underwent hematopoietic stem cell transplantation (HSCT) and had a fatal outcome.

## CASE REPORT

Five-year-old male patient from the district of Independencia in Lima, Peru, referred to the Instituto Nacional de Salud del Niño San Borja with a diagnosis of very severe bone marrow aplasia, paroxysmal nocturnal hemoglobinuria (PNH) clone, no response to immunosuppressive therapy (IST) and no HLA (human leukocyte antigens) compatible donor. In addition, the patient had received multiple transfusions and reported no allergies or adverse drug reactions.

The patient underwent a first haploidentical HSCT, TCR (T-cell receptor) α/β - CD19 -), conditioning regimen with fludarabine, antithymocyte globulin rabbit, cyclophosphamide, and total body irradiation. However, the transplant failed and, subsequently, he developed persistent febrile neutropenia, requiring broad-spectrum antimicrobial treatment and even had to undergo surgery due to acute appendicitis. The patient evolved favorably and three months after the first HSCT, a second haploidentical HSCT was performed, which resulted in graft failure; he then had profound neutropenia (absolute neutrophil count (ANC) = 0 cells/µL) with episodes of intermittent fever and partial response to broad-spectrum antimicrobial regimens for neutropenic colitis, central venous catheter-associated infection and probable invasive pulmonary aspergillosis, positive serum galactomannan, 2.1 optical density (OD).

On day 110, after HSCT, and after episodes of persistent fever and with elevated C-reactive protein values, the patient was afebrile for several days and reached the lowest C-reactive protein value in weeks of antimicrobial therapy (C-reactive protein = 30 mg/L, normal value = < 5mg/L), receiving meropenem, ciprofloxacin, linezolid and voriconazole for a prolonged period.

Subsequently, it was decided to progressively withdraw the antimicrobials, but he presented fever again, C-reactive protein values increased progressively and, from day 140, renal function deterioration was exacerbated (creatinine: 3.17 mg/dL; urea: 117 mg/dL; urea nitrogen: 54.64 mg/dL), with bilateral eyelid swelling and oral mucositis. Despite restarting antimicrobials, intense abdominal pain appeared on day 146 post- HSCT, predominantly in the epigastrium, as well as disseminated reddish maculopapular lesions on the skin, and pain in the right knee without evidence of phlogosis, which increased with extension movements. Due to abdominal involvement, the patient did not continue receiving oral prophylaxis with posaconazole and, due to clinical deterioration, treatment with caspofungin started empirically (day 147) at a dose of 70 mg/SC/day (loading dose), then 50 mg/SC/day. According to laboratory results, the patient persisted with ANC=0 cells/µL, C-reactive protein increased to 180 mg/dL, galactomannan study was negative (0.08 OD), viral loads for cytomegalovirus (CMV), adenovirus (ADV), and Epstein-Barr virus (EBV) were undetectable.

Four sets of blood cultures were taken (including peripheral blood, proximal and distal lumen transcatheter), the first three were negative but the last one (transcatheter blood) was obtained on day 147, three days after the sample was taken, and showed abundant pseudohyphae in blood from the proximal and distal lumen catheter, *Saprochaete capitata*/*Magnusiomyces capitatus* were identified in that sample. It is worth mentioning that the central venous catheter did not show signs of phlogosis in the insertion area, but it had a prolonged duration (57 days) and was not removed. Other laboratory tests at the time of diagnosis are shown in Table 1. The patient died of septic shock (day 150) on the same day that the identification of the causative agent of the invasive fungal infection was reported.

**Table 1:** Laboratory tests at the time of diagnosis of *S. capitata*

Variable	Reference	Result
Hemoglobin (g/dL)	11,5 - 14,5	9,9
Platelets (10^3^ per mm^3^)	150 - 350	37
Leucocytes (10^3^ per mm^3^)	4,5 - 13,5	0,02
Segmented neutrophils (%)	32 - 54	0
Lymphocytes (%)	28 - 48	100
GPT (U/L)	0 - 29	19
GOT (U/L)	0 - 48	64
Bilirubin total (mg/dL)	0,1 - 1	0,21
Creatinine (mg/dL)	0,32 - 0,59	2,65
Urea (mg/dL)	15 - 36	102
PCR (mg/dL)	0,0 - 0,5	180

GPT: glutamic pyruvic transaminase. GOT: glutamic oxaloacetic transaminase. CRP: C-reactive protein.

## DISCUSSION

Invasive fungal infections by rare fungi constitute a worrisome and emerging situation, with high mortality and different patterns of antifungal resistance depending on the species ^7^. In Peru, there are few reports of invasive fungal infections by rare fungi, both yeast and filamentous ^8^ ^,^ ^9^. *S. capitata* is a rare yeast-like fungus that has not been previously described in Peru as a cause of invasive fungal infection in humans and has been identified mainly in immunocompromised patients, as described in the present case of a patient diagnosed with bone marrow aplasia who underwent HSCT.

The epidemiology of *S. capitata* infections is still not well established and the incidence is low, due to limitations in microbiological diagnostic and epidemiological surveillance methods, which also differ between countries ^1^ ^,^ ^7^. Most nosocomial cases and outbreaks have been described mainly in European countries ^10^, while in Latin America, Brazil has reported cases in patients with acute myeloid leukemia ^11^ ^,^ ^12^.

Recently, the European Confederation for Medical Mycology (ECMM) in cooperation with the International Society for Human and Animal Mycology (ISHAM) and the American Society for Microbiology (ASM) published a guideline for the diagnosis and management of rare yeast infections, which includes infections by *Saprochaete* or *Magnusiomyces* spp ^13^. The initial diagnosis should be made from the isolation of the fungus in a culture medium obtained from a sterile site of the body, such as blood cultures. In our case report, the fungus was identified in transcatheter blood cultures using the Phoenix automated system ^12^ ^,^ ^13^.

Regarding treatment, the antifungal of choice is amphotericin B associated or not with flucytosine or voriconazole (moderate recommendation), and it is recommended to avoid the use of echinocandins, especially as monotherapy, and there have even been reported cases of gap fungal infection by *S. capitata* in patients receiving echinocandins ^13^ ^,^ ^14^. Likewise, echinocandins and fluconazole have high values of *in vitro* minimum inhibitory concentrations (MIC), compared to MIC values for other antifungals such as voriconazole (0.03-1 mg/L), amphotericin B (0.03-2 mg/L), itraconazole (0.01-1 mg/L) and posaconazole (0.03-1 mg/L), flucytosine (0.06-64 mg/L) ^6^. Additionally, the guideline highlights the need for central venous catheter removal (strong recommendation) ^13^. In this case, the patient had to stop receiving antifungal prophylaxis with posaconazole due to gastrointestinal manifestations; treatment with caspofungin continued, which is not an alternative in the treatment of *S. capitata*. Amphotericin B was not used due to the increase in creatinine and urea values (renal involvement). In addition, although there were no signs of inflammation at the central venous catheter insertion site, the catheter was not changed or removed, despite the prolonged stay.

One of the limitations of our case report was that it was not possible to obtain images of the fungus identified in the blood culture which was typified with an automated method; additionally, it was not possible to perform complementary microbiological studies such as MALDI-TOF (Matrix-Assisted Laser Desorption/Ionization - Time-Of-Flight) and/or molecular tests or antifungogram. Likewise, imaging studies such as tomography and ultrasound were not included, since they are more than a month apart from the reported infectious event.

In conclusion, invasive fungal infection by *S. capitata* is a rare but emerging and potentially fatal infection, especially in immunocompromised patients, and in those with persistent febrile neutropenia, and prolonged use of invasive devices such as central venous catheters. Improving and implementing diagnostic tools for the timely identification of fungal infections during clinical practice is key to establish early diagnosis in order to start targeted antifungal therapy that can prevent the progression and fatal outcome of this disease.
