# Supplementary material for: Invasive fungal infection by Saprochaete capitata in a child with bone marrow aplasia
Source: Rev Peru Med Exp Salud Publica. 2022 Sep 30;39(3):372–5. doi: 10.17843/rpmesp.2022.393.10955 (PMC11397778; doi:10.17843/rpmesp.2022.393.10955)
Supplement: Translation

# Translation: Infección fúngica invasiva por *Saprochaete capitata *en un niño con aplasia medular

**Authors:** Julio Maquera-Afaray, Camila Escajadillo-Vergara, Jeanpiero Chire-Mercado, María Paula Durand

**Affiliations:** 1 Instituto Nacional de Salud del Niño San Borja, Lima, Perú.; 2 Universidad Privada de Tacna, Tacna, Perú.

**Keywords:** Infecciones Fúngicas Invasoras, Micosis, Hongos, Antifúngicos, Anemia Aplásica

## Abstract

*Saprochaete capitata* es una causa rara de infección fúngica invasiva en pacientes inmunocomprometidos con alta mortalidad y resistencia antifúngica. Presentamos el caso de un niño de cinco años con diagnóstico de aplasia medular, sometido a trasplante de progenitores hematopoyéticos (TPH), que cursó con neutropenia febril persistente, dolor abdominal intenso, aparición de lesiones maculopapulares en piel y deterioro de la función renal. Se identificó la presencia de *S. capitata*, en hemocultivos transcatéter venoso central. Esta infección fúngica invasiva resulta ser rara, pero emergente y potencialmente mortal, en pacientes con neutropenia febril persistente y uso prolongado de dispositivos invasivos intravasculares como catéter venoso central.

## INTRODUCCIÓN

*Saprochaete capitata *(anamorfo)/*Magnusiomyces capitatus *(telemorfo), previamente conocido como* Geotrichum capitatum o Blastoschizomyces capitatus,* es un hongo levaduriforme clasificado en la familia Dipodascaceae, orden Saccharomycetales ^1^. Este microorganismo se encuentra en el medioambiente, especialmente en el suelo, y puede colonizar la piel, tracto respiratorio y gastrointestinal en los seres humanos ^2^. Asimismo, *S. capitata* es un hongo oportunista que afecta principalmente a pacientes inmunocomprometidos con enfermedad oncohematológica y neutropenia prolongada ^3^. 

La tasa incidencia de fungemia por *S. capitata* es baja, siendo considerada una causa de infección fúngica invasiva rara, con reportes de casos esporádicos y algunos brotes nosocomiales descritos en diferentes países ^1^ ^,^ ^4^; no obstante, en las últimas décadas se ha incrementado el número de casos de *S. capitata*, debido al mayor uso de terapias inmunosupresoras e identificación del agente causal por el uso de mejores métodos diagnósticos microbiológicos ^2^ ^,^ ^5^. A pesar de ello, la mortalidad descrita por *S. capitata* todavía es alta, fluctuando entre 40 a 75% de los casos ^1^ ^,^ ^6^.

Mediante el presente artículo reportamos el primer caso de infección fúngica invasiva por *S. capitata* en el Perú, que se presentó en un niño inmunocomprometido con aplasia medular sometido a trasplante de progenitores hematopoyéticos (TPH), y que tuvo un desenlace fatal. 

## REPORTE DE CASO

Paciente varón de cinco años, procedente del distrito de Independencia en Lima, Perú, referido al Instituto Nacional de Salud del Niño San Borja con diagnóstico de aplasia medular muy severa, clona de hemoglobinuria paroxística nocturna (HPN), sin respuesta a terapia inmunosupresora (TIS) y sin donante compatible HLA (del inglés, *human leukocyte antigens*). Además, se registraron como antecedentes haber recibido politransfusiones y no reportar alergias ni reacciones adversas a medicamentos. 

El paciente fue sometido a un primer TPH tipo haploidéntico, TCR (del inglés *T-cell receptor*) α/β - CD19 -), régimen de acondicionamiento con fludarabina, globulina antitimocítica de conejo, ciclofosfamida e irradiación corporal total. Sin embargo, el trasplante fracasó y, posteriormente, cursó con neutropenia febril persistente, requiriendo tratamiento antimicrobiano de amplio espectro e, incluso, tuvo que ser intervenido quirúrgicamente por presentar apendicitis aguda. El paciente evolucionó favorablemente y tres meses después del primer TPH, se realizó un segundo TPH haploidéntico que también fracasó por falla en el prendimiento y cursó con neutropenia profunda (recuento absoluto de neutrófilos (RAN) = 0 células/µL), con episodios de fiebre intermitente y respuesta parcial a los esquemas de antimicrobianos de amplio espectro empleados por colitis neutropénica, infección asociada a catéter venoso central y aspergilosis pulmonar invasiva probable, galactomanano sérico positivo, 2,1 densidad óptica (DO).

En el día 110, luego del TPH, tras episodios de fiebre persistente y con valores elevados de proteína C reactiva, el paciente estuvo afebril por varios días y alcanzó el valor de proteína C reactiva más bajo en semanas de terapia antimicrobiana (proteína C reactiva = 30 mg/L, valor normal = < 5mg/L), recibiendo meropenem, ciprofloxacino, linezolid y voriconazol por tiempo prolongado. 

Posteriormente, se decidió el retiro progresivo de los antimicrobianos, pero nuevamente presentó fiebre, los valores de proteína C reactiva se incrementaron progresivamente y, desde el día 140, se exacerbó el deterioro de la función renal (creatinina: 3,17 mg/dL; urea: 117 mg/dL; nitrógeno ureico: 54,64 mg/dL), con edema bipalpebral y mucositis oral. A pesar de la reintroducción de antimicrobianos, en el día 146 pos-TPH se agregó dolor abdominal intenso a predominio de epigastrio, aparecieron lesiones maculopapulares diseminadas en piel de aspecto rojizo, y dolor en rodilla derecha sin evidencia de signos de flogosis, pero que se incrementaba al realizar movimientos de extensión. Por el compromiso abdominal el paciente no continuó recibiendo profilaxis vía oral con posaconazol y, debido al deterioro clínico, se inició de forma empírica caspofungina (día 147) a dosis 70 mg/SC/día (dosis de carga), luego 50 mg/SC/día. Según los resultados de laboratorio, el paciente persistió con RAN=0 células/µL, la proteína C reactiva se incrementó hasta 180 mg/dL, el estudio de galactomanano fue negativo (0,08 DO), las cargas virales para citomegalovirus (CMV), adenovirus (ADV), y el virus de Epstein-Barr (VEB) fueron no detectables. 

Se realizó la toma de cuatro sets de hemocultivos (que incluyeron sangre periférica, transcatéter de lumen proximal y distal), los tres primeros fueron negativos y el último tomado (solo de sangre transcatéter) en el día 147, tres días después de la toma de la muestra, se identificó pseudohifas abundantes en sangre de catéter de lumen proximal y distal, con identificación de *Saprochaete capitata/Magnusiomyces capitatus*, cabe mencionar que en el catéter venoso central no se evidenció signos de flogosis en zona de inserción, pero que tenía una duración prolongada (57 días) y no fue removido. Otros exámenes de laboratorio al momento del diagnóstico se muestran en la Tabla 1. El paciente falleció por choque séptico (día 150), el mismo día que se reportó la identificación del agente causal de infección fúngica invasiva.

**Tabla 1:** Exámenes de laboratorio al momento del diagnóstico*.*

Variable	Rango referencial	Resultado
Hemoglobina (g/dL)	11,5 - 14,5	9,9
Plaquetas (10^3^ por mm^3^)	150 - 350	37
Leucocitos (10^3^ por mm^3^)	4,5 - 13,5	0,02
Segmentados (%)	32 - 54	0
Linfocitos (%)	28 - 48	100
TGP (U/L)	0 - 29	19
TGO (U/L)	0 - 48	64
Bilirrubina total (mg/dL)	0,1 - 1	0,21
Creatinina (mg/dL)	0,32 - 0,59	2,65
Urea (mg/dL)	15 - 36	102
PCR (mg/dL)	0,0 - 0,5	180

## DISCUSIÓN

Las infecciones fúngicas invasivas por hongos raros constituyen una situación preocupante y emergente, con alta mortalidad y diferentes patrones de resistencia antifúngica según la especie ^7^. En Perú son pocos los reportes realizados por infecciones fúngicas invasivas por hongos raros, tanto levaduriformes como filamentosos ^8^ ^,^ ^9^. *S. capitata* es un hongo raro levaduriforme que no ha sido previamente descrito en el Perú como causa de infección fúngica invasiva en humanos y que ha sido identificado, principalmente, en pacientes inmunocomprometidos, como se describe en el presente caso en un paciente con diagnóstico de aplasia medular y sometido a TPH. 

La epidemiología de las infecciones por *S. capitata* todavía no está bien establecida y la incidencia es baja, debido a las limitaciones existentes en los métodos de diagnóstico microbiológico y vigilancia epidemiológica, que también difieren entre países ^1^ ^,^ ^7^. La mayoría de los casos y brotes nosocomiales se han descrito principalmente en países de Europa ^10^, mientras que, en Latinoamérica, Brasil ha reportado casos en pacientes con leucemia mieloide aguda ^11^ ^,^ ^12^. 

Recientemente, la European Confederation for Medical Mycology (ECMM) en cooperación con International Society for Human and Animal Mycology (ISHAM) y la American Society for Microbiology (ASM) publicaron una guía para el diagnóstico y manejo de las infecciones por hongos levaduriformes raros, que incluye infecciones por *Saprochaete* o *Magnusiomyces* spp ^13^. El diagnóstico inicial se obtiene del aislamiento del hongo en un medio de cultivo de una muestra procedente de un sitio estéril del cuerpo, como son los hemocultivos, en el presente reporte de caso la identificación del hongo se obtuvo en hemocultivos transcatéter mediante el sistema automatizado Phoenix ^12^ ^,^ ^13^. 

En relación con el tratamiento, el antifúngico de elección es la anfotericina B asociada o no con flucitosina o voriconazol (recomendación moderada), y se recomienda evitar el uso de equinocandinas, especialmente como monoterapia e, incluso, se han reportado casos de infección fúngica de brecha por *S. capitata* en pacientes que reciben equinocandinas ^13^ ^,^ ^14^. Asimismo, las equinocandinas y fluconazol presentan valores altos de concentraciones mínimas inhibitorias (CMI) *in vitro*, en comparación con los valores de CMI para los otros antifúngicos como voriconazol (0,03-1 mg/L), anfotericina B (0,03-2 mg/L), itraconazol (0,01-1 mg/L) y posaconazol (0,03-1 mg/L), flucitosina (0,06-64 mg/L) ^6^. Adicionalmente, la guía destaca la necesidad del retiro de catéter venoso central (recomendación fuerte) ^13^. En el caso, el paciente tuvo que dejar de recibir profilaxis antifúngica con posaconazol debido a las manifestaciones gastrointestinales que presentó, se continuó con caspofungina que no constituye una alternativa en el tratamiento de *S. capitata*. No se empleó anfotericina B por el incremento de los valores de creatinina y urea (compromiso renal). Además, aunque no se evidenció signos de inflamación en la zona de inserción del catéter venoso central, este no fue cambiado o removido, a pesar de la permanencia prolongada.

Cabe mencionar como limitaciones en el presente reporte de caso que no se logró obtener imágenes del hongo identificado en el hemocultivo cuya tipificación fue a través de un método automatizado no se pudo realizar estudios complementarios microbiológicos como MALDI-TOF (del inglés *Matrix-Assisted Laser Desorption/Ionization - Time-Of-Flight*) y/o pruebas moleculares ni antifungograma. Asimismo, no se han consignado estudios de imágenes como tomografía y ecografía, debido que distan en más de un mes del evento infeccioso reportado, por lo cual no fueron considerados. 

En conclusión, la infección fúngica invasiva por *S. capitata* constituye una infección rara, pero emergente y potencialmente mortal, en especial en pacientes inmunocomprometidos, neutropenia febril persistente y uso prolongado de dispositivos invasivos como catéter venoso central. Mejorar e implementar herramientas diagnósticas para la identificación oportuna de infecciones fúngicas en la práctica clínica es clave para establecer un diagnóstico e inicio temprano de la terapia antifúngica dirigida que pueda evitar la progresión y desenlace fatal de la enfermedad.
